# Multi-source remote sensing-based landslide investigation: the case of the August 7, 2020, Gokseong landslide in South Korea

**DOI:** 10.1038/s41598-024-59008-4

**Published:** 2024-05-27

**Authors:** Shin-Kyu Choi, Ryan Angeles Ramirez, Hwan-Hui Lim, Tae-Hyuk Kwon

**Affiliations:** 1 Structural and Seismic Technology Group, Korea Electric Power Research Institute (KEPRI), Daejeon, 34056 Republic of Korea; 2 Department of Civil Engineering, University of Santo Tomas (UST), 1008 Manila, Philippines; 3 Department of Civil and Environmental Engineering, Korea Advanced Institute of Science and Technology (KAIST), Daejeon, 34141 Republic of Korea

**Keywords:** Natural hazards, Engineering

## Abstract

Landslides pose a growing concern worldwide, emphasizing the need for accurate prediction and assessment to mitigate their impact. Recent advancements in remote sensing technology offer unprecedented datasets at various scales, yet practical applications demand further case studies to fully integrate these technologies into landslide analysis. This study presents a case study approach to fully leverage variety of multi-source remote sensing technologies for analyzing the characteristics of a landslide. The selected case is a landslide with a long runout debris flow that occurred in Gokseong County, South Korea, on August 7, 2020. The chosen multi-source technologies encompass digital photogrammetry using RGB and multi-spectral imageries, 3D point clouds acquired by light detection and ranging (LiDAR) mounted on an unmanned aerial vehicle (UAV), and satellite interferometric synthetic aperture radar (InSAR). The satellite InSAR analysis identifies the initial displacement, triggered by rainfall and later transforming into a debris flow. The utilization of digital photogrammetry, employing UAV-RGB and multi-spectral image data, precisely delineates the extent affected by the landslide. The landslide encompassed a runout distance of 678 m, featuring an initiation zone characterized by an average slope of 35°. Notably, the eroded and deposited areas measured 2.55 × 10^4^ m^2^ and 1.72 × 10^4^ m^2^, respectively. The acquired UAV-LiDAR data further reveal the eroded and deposited landslide volumes approximately measuring 5.60 × 10^4^ m^3^ and 1.58 × 10^4^ m^3^, respectively. This study contributes a valuable dataset on a rainfall-induced landslide with a long runout debris flow, underscoring the effectiveness of multi-source remote sensing technology in monitoring and comprehending complex landslide events.

## Introduction

Landslides refer to sudden collapse and rapid downstream movement of destabilized earth ground, which can be primed or triggered by various factors, including rainfall, earthquakes, and human activities. These events are highly unpredictable, and they carry immense velocity and impact force, posing significant hazards. Several catastrophic landslide-related damages have been reported around the world, such as the Woomyeon landslide in Seoul^1–3^, the Montecito landslide in California^4,5^, the Mabian landslide in Mabian County^6^, the Livadea landslide in Livadea village^7^, the Jichang landslide in Shuicheng County^8^, and the Aniangzhia landslide in Danba County^9^. As heavy rains become more concentrated in localized regions, the frequency and severity of landslide hazards are becoming increasingly pronounced in numerous countries.

Records on past landslide events are one of the critical ingredients to build a capacity for accurate prediction of potential landslides. The landslide record or landslide inventory needs to include the volumes of initial source and final deposited mass, and landslide characteristics (e.g., rheology, soil properties, erosion rate) as well as the geographic, geologic and topographic data. Hence, conducting a comprehensive investigation of landslide events becomes crucial, involving a quantitative assessment of their geometry, such as area, volume, and runout distance, along with other relevant landslide-related characteristics. In general, walk-in field surveys immediately after a landslide event can provide valuable information^10–18^. However, field visits are often restricted due to the safety concern, such as a potential danger of progressive collapse as an example.

Recently, remote sensing technology has emerged as a valuable tool to overcome this limitation as it can effectively monitor hard-to-reach areas and conduct prolonged and periodic observations. Additionally, it is cost-effective, time-saving, and portable. The types of remote sensing technology are classified according to the sensors (or cameras) mounted on UAVs (i.e., optical camera, LiDAR sensor, and radar sensor). Optical data typically includes visible radiation (red, green, and blue bands; RGB data) as well as infrared radiation (IR) range. In addition, monitoring using satellite radio detection and ranging (radar) enables observation of tiny displacements at the millimeter scale and can also observe past displacement histories. Therefore, the remote sensing techniques are widely utilized not only in the field of landslide disasters but also in various geo-science fields which requires long-term monitoring over a large area^19–26^.

Use of a single technique often poses a challenge in landslide surveys. For example, the optical imaging, as a passive method, is difficult to acquire topographic information in densely forested areas due to the occlusion effect^27–29^. Although the 3D point clouds gathered from LiDAR can provide topographic information, its lack of RGB information limits the object identification. The satellite radar is highly effective in detecting tiny displacements before a landslide occurs. However, its capability to observe meter-scale displacements with massive earth movements is limited. Rather than using a single technique, integration of multiple remote sensing technologies offers a promising approach to effective landslide monitoring^8,30–35^.

This study presents a comprehensive investigation on a landslide, focusing on the detailed analysis of its characteristics through the integration of diverse remote sensing technologies. The chosen case pertains to a landslide with a long runout debris flow that occurred in Gokseong County, South Korea, on August 7, 2020. A suite of multi-source technologies was strategically employed, including digital photogrammetry utilizing RGB and multi-spectral imagery, 3D point clouds derived from light detection and ranging (LiDAR) mounted on an unmanned aerial vehicle (UAV), and satellite interferometric synthetic aperture radar (InSAR). In particular, InSAR technology facilitated the detection of landslide initiation, while RGB and multi-spectral information aided in delineating the extent of the affected areas. Additionally, for precise quantification of landslide magnitude, 3D LiDAR point clouds were utilized to compute the volumes involved. Through the synergistic utilization of these diverse remote sensing technologies, this study aims to elevate the precision and efficacy of landslide investigations.

## Study area

**Figure 1:**
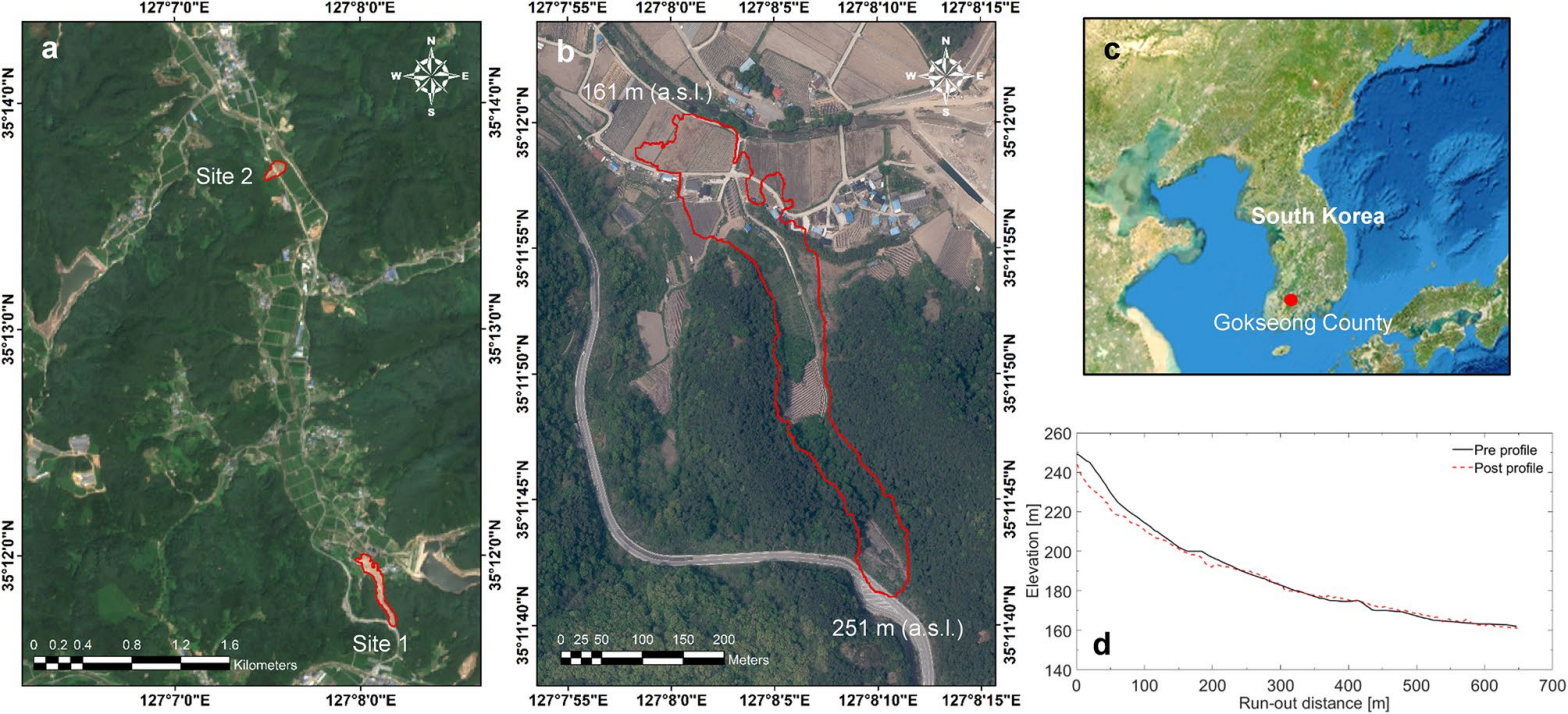
Optical images of the Gokseong landslide site: (**a**) Sentinel-2 image after the event in Sites 1 and 2, (**b**) before the event in Site 1 (captured by Korea National Geographic Information Institute, KNGII in 2019), (**c**) The image representing the location of Gokseong County in South Korea and (**d**) the profiles before and after the occurrence of the landslide event. Note that the areas highlighted by the red polygons indicate the landslide areas.

**Figure 2:**
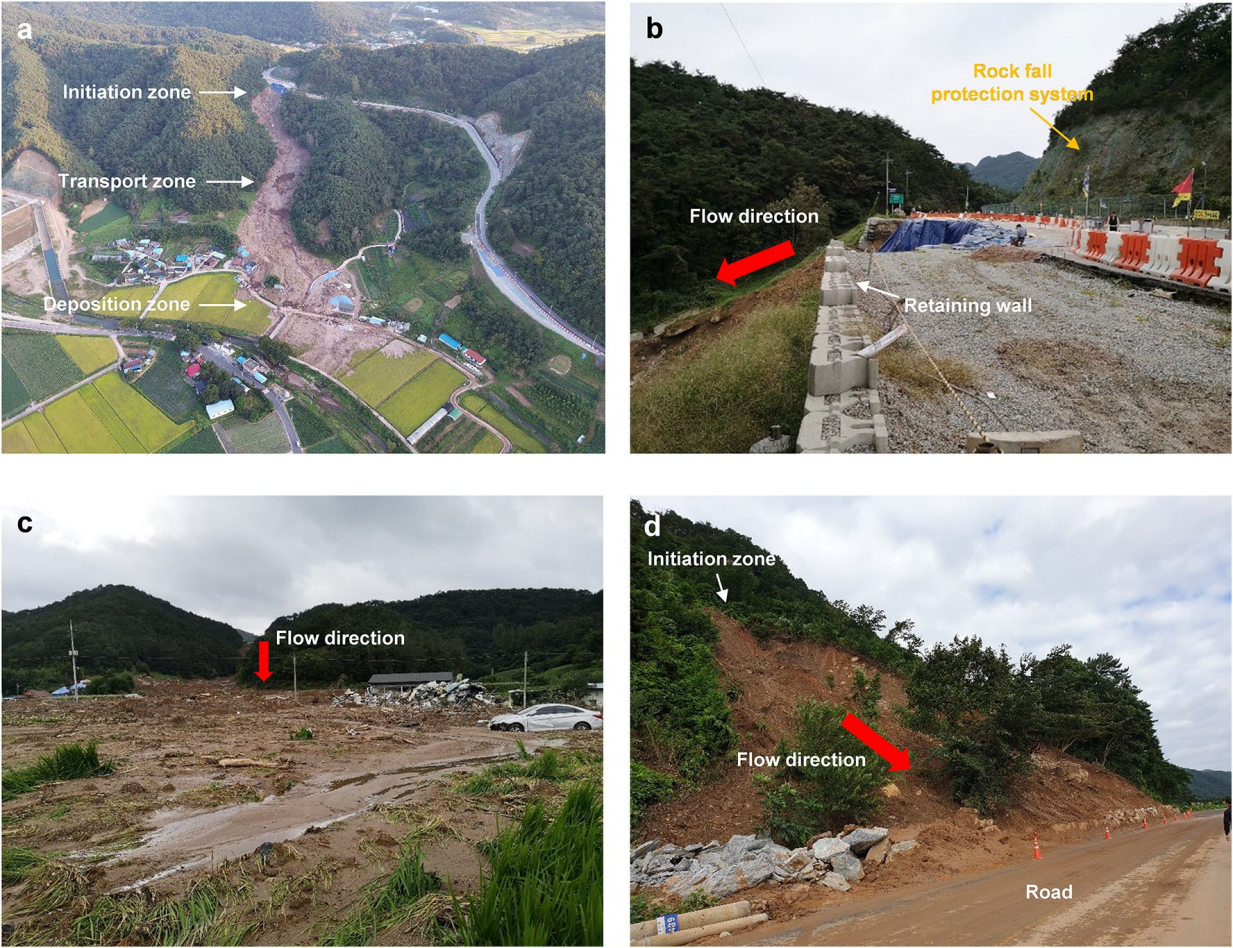
Digital photographs of the Gokseong landslide: (**a**) Overview of the landslide (Site 1), (**b**) the initiation zone of Site 1, (**c**) the deposition zone of Site 1, and (**d**) overview of the landslide (Site 2).

On August 7, 2020, a catastrophic landslide occurred at approximately 8:30 p.m. on a mountain behind a village in Osan town, Gokseong County, South Jeolla Province, South Korea (35°11′40″ N, 127°8′10″ E; Fig. 1), referred to as the Gokseong landslide. Figure 1d represents the elevation profiles of the landslide channel before and after the event. It is a typical form of debris flows where eroded (or collapsed) sediment from the upstream area travels a long distance and accumulates in the downstream area. The primary trigger for this landslide was three consecutive days of heavy rainfall. The event caused extensive devastation to the downstream village as a significant volume of debris traveled a considerable distance, resulting in five fatalities, five houses buried, and a section of road collapsed (Fig. 2). Approximately 30 residents residing near the landslide site were evacuated. Five days post the landslide event, this study conducted a UAV field survey.

**Figure 3:**
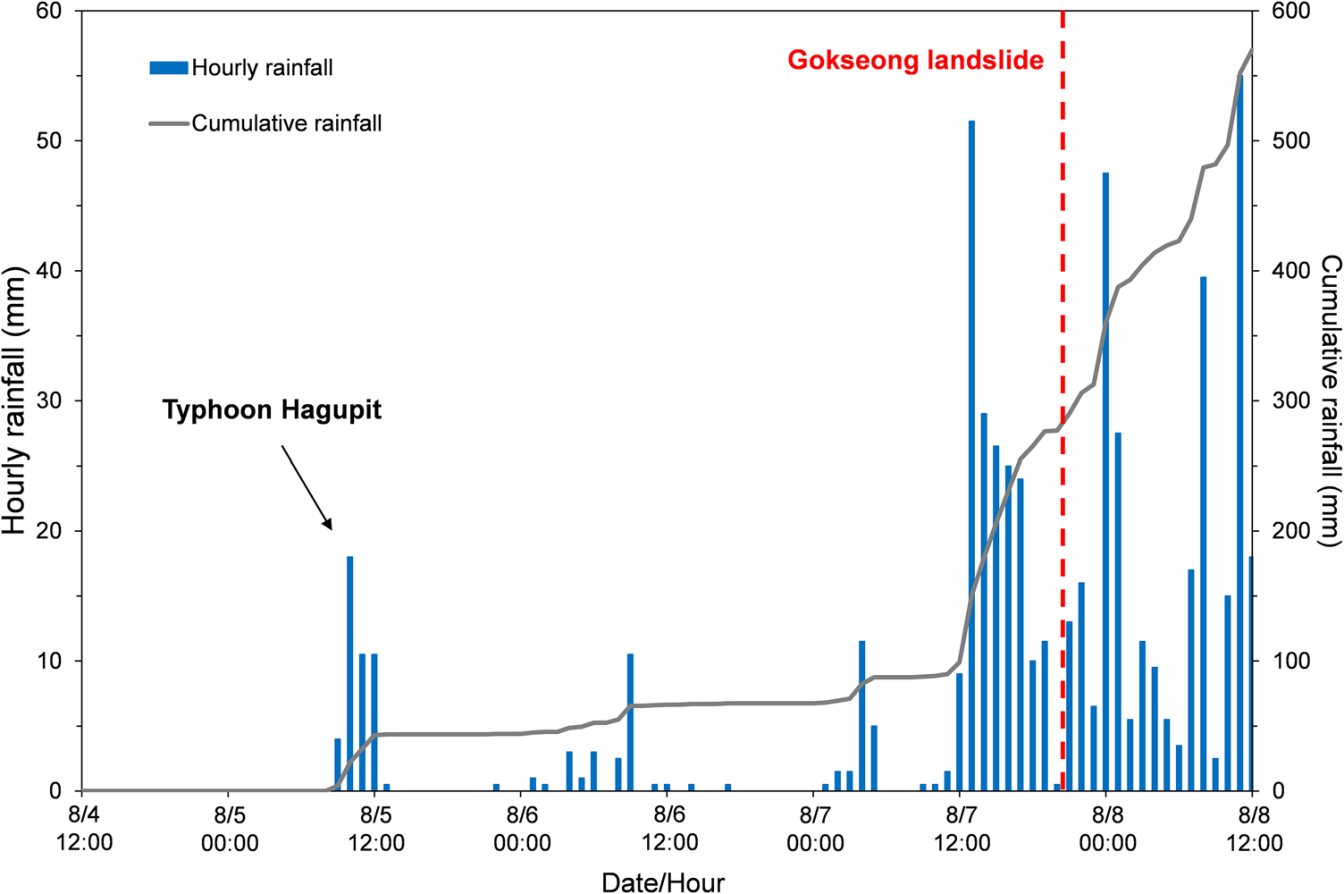
Hourly and cumulative rainfalls before the Gokseong landslide (at approximately 8:30 p.m. on August 7th).

South Korea exhibits intricate climatic patterns arising from the interplay of continental and oceanic influences, featuring an average annual precipitation of 1,190 mm. The monsoon season, extending from July to September, contributes to over 50% of the total annual rainfall. Figure 3 presents rainfall data from a local meteorological station located 6 km from the landslide site, sourced from the Korea Meteorological Administration (KMA). The precipitation graph highlights the commencement of intense rainfall around 8:30 a.m. on August 5, 2020, two days before the landslide event. Approximately 7.5 h before the landslide occurrence, cumulative rainfall had surpassed 150 mm, with the maximum hourly rainfall recorded at 51.5 mm. The antecedent cumulative rainfall in the three days leading up to the landslide event amounted to 290 mm (Fig. 3). Additionally, on August 5, 2020, Typhoon Hagupit induced heavy rainfall in the region.

## Materials and methods

Landslide monitoring involves distinct phases before and after the occurrence. Before a landslide event, it is important to conduct ongoing monitoring by regularly measuring displacement in areas prone to such risks. Employing UAVs for this purpose proves to be inefficient. However, utilizing satellites, despite longer monitoring intervals, offers an effective alternative. After a landslide, quantitative assessments to area, volume, changes in elevation are required to identify triggers and formulate an effective recovery plan. Given that landslides typically occur within a range of several meters to hundreds of meters, the use of LiDAR data is more appropriate than radar data. Prior to the landslide, the satellite InSAR technology was utilized to detect any indications of pre-failure movement. Subsequent to the landslide event, the volumes of eroded and deposited materials were calculated using topographic data obtained from the 3D LiDAR sensor. Additionally, RGB and multi-spectral data were used to estimate the extent of the landslide damage area.

### Pre-failure monitoring using satellite SAR data

**Figure 4:**
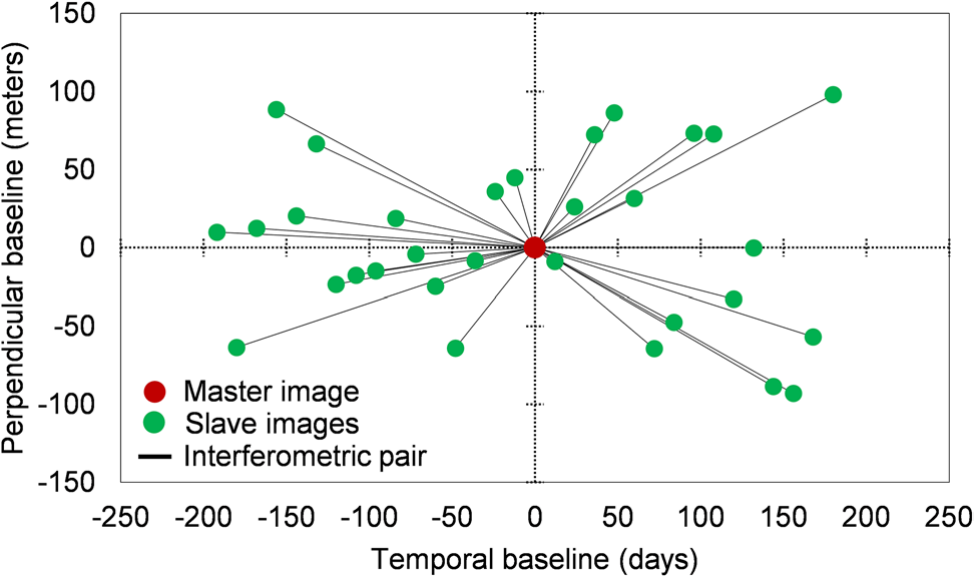
Pairing of master and slave synthetic aperture radar (SAR) images.

This study involved the collection and processing of 32 satellite SAR data from the ascending Sentinel-1 mission, as shown in Fig. 4. The dataset covered the period from August 1, 2019 to August 7, 2020, including the pre-failure state. The InSAR stack overview operator of the Sentinel Application Platform (SNAP) automatically selected the master image (January 1, 2020 in this analysis). Subsequently, the remaining images were co-registered as slave images to match the geometry of the master image. Figure 4 illustrates the spatiotemporal distribution of the Sentinel-1 SAR data stack and the interferometric pairs used in this study. The satellite InSAR method is capable of providing near-real-time monitoring of ground displacement, overcoming temporal, spatial, and meteorological constraints. Time-series InSAR analysis using multi-temporal satellite SAR effectively detects tiny displacements over a long period. In particular, we employed the permanent scatterer InSAR (PS-InSAR) method^36^, which is one of reliable and thus widely used time-series InSAR analysis methods. The PS-InSAR observes temporal deformation by using ground targets that exhibit stable phase behavior over the satellite radar data stack. The targets are primarily observed in in urban areas such as buildings, maintaining stable coherence and experiencing minimal noise interference. Compared to other InSAR analysis methods, it exhibits fewer atmospheric errors, enabling more precise estimation of ground displacement. Furthermore, it facilitates the analysis of long-term temporal deformation. The PS-InSAR analysis was carried out through a semi-automated processing chain with a two-stage workflow, consisting of the single master differential InSAR processing and the time series analysis.

### Landslide area mapping using optical data

RGB and multi-spectral images were acquired for digital photogrammetry to examine the geometric characteristics of the landslide and analyze the affected area. The RGB images were captured using an optical digital camera (X5S, DJI) mounted on the DJI Inspire 2 UAV. Additionally, a multi-spectral digital camera (RedEdge-MX, MicaSense), capable of capturing five bands (i.e., 475 nm ± 32 nm for the blue band, 560 nm ± 27 nm for the green band, 668 nm ± 14 nm for the red band, 717 nm ± 12 nm for red edge band, and 842 nm ± 57 nm for near-infrared (NIR) band), was installed on the DJI Inspire 2 UAV to obtain multi-spectral images. For data analysis, 3D point clouds were generated from overlapped images taken from various locations using the structure from motion algorithm (SfM) with the Agisoft Metashape program (v.1.5.5). Ground control points (GCP) were employed to ensure high accuracy in obtaining point clouds, as the global navigation satellite system (GNSS) sensor mounted on the UAV had limited accuracy.

In particular, this study employed the normalized difference vegetation index (NDVI) to delineate landslide-affected areas^37,38^, and it is calculated using the NIR and red band reflectance, as follows:

1 $$NDVI = \frac{{(R_{NIR} - R_{red} )}}{{(R_{NIR} + R_{red} )}}$$

where R_NIR_ is the reflectance of the NIR band and R_red_ is the reflectance of the red band. The NDVI proves more accurate than results derived from RGB images, particularly in forested and vegetated areas, common locations for landslides. Its application extends to extracting landslide-affected areas, considering diverse characteristics contingent on land cover types. In this study, the NDVI was used to differentiate various land-cover types, with vegetated areas exhibiting higher NDVI values, while non-vegetated regions, such as soil or concrete, showed lower values. Therefore, when a landslide occurs, the NDVI decreases significantly as trees and vegetation are uprooted, leaving only exposed soil behind^38^. Leveraging these distinctive features, the occurrence of landslides was analyzed using multi-spectral data at the Gokseong landslide site.

### Topographic change estimation using LiDAR data

The landslide volume plays a crucial role in back-analyzing the flow characteristics of landslides. Additionally, post-disaster recovery planning necessitates volume information, which can be derived from changes in elevation obtained through remote sensing. This study estimated the landslide volume based on the change in topographic elevation before and after the landslide, where a UAV-LiDAR system was used to obtain the topographic information. The system was composed of a UAV (Matrice 600 Pro, DJI), GNSS, inertial measurement unit (IMU), LiDAR sensor (VLP-16, Velodyne), and other components. Detailed information on the UAV-LiDAR system used in this study can be found in Choi et al.^39^, including its configuration, calibration, and accuracy. The UAV-LiDAR system was flown at an altitude of 300 m with a velocity of 3 m/s to acquire a 3D LiDAR point cloud of the area after the landslide event. Then, the topographic change was quantified by using the multiscale model-to-model cloud comparison (M3C2) method, which calculates the distance between two point clouds even in cases where homologous parts are not explicitly defined^40^. When two point clouds are produced, the normal vector is determined by analyzing the points within the circle defined by the user. The normal vector indicates the direction of change between the two point clouds. Next, the average elevation is determined by analyzing the points within a cylinder defined by the user. This entire process is repeated for each point separated by the input distance, allowing for a comprehensive analysis of topographic changes between the two point clouds.

## Results

### Landslide pre-failure analysis

**Figure 5:**
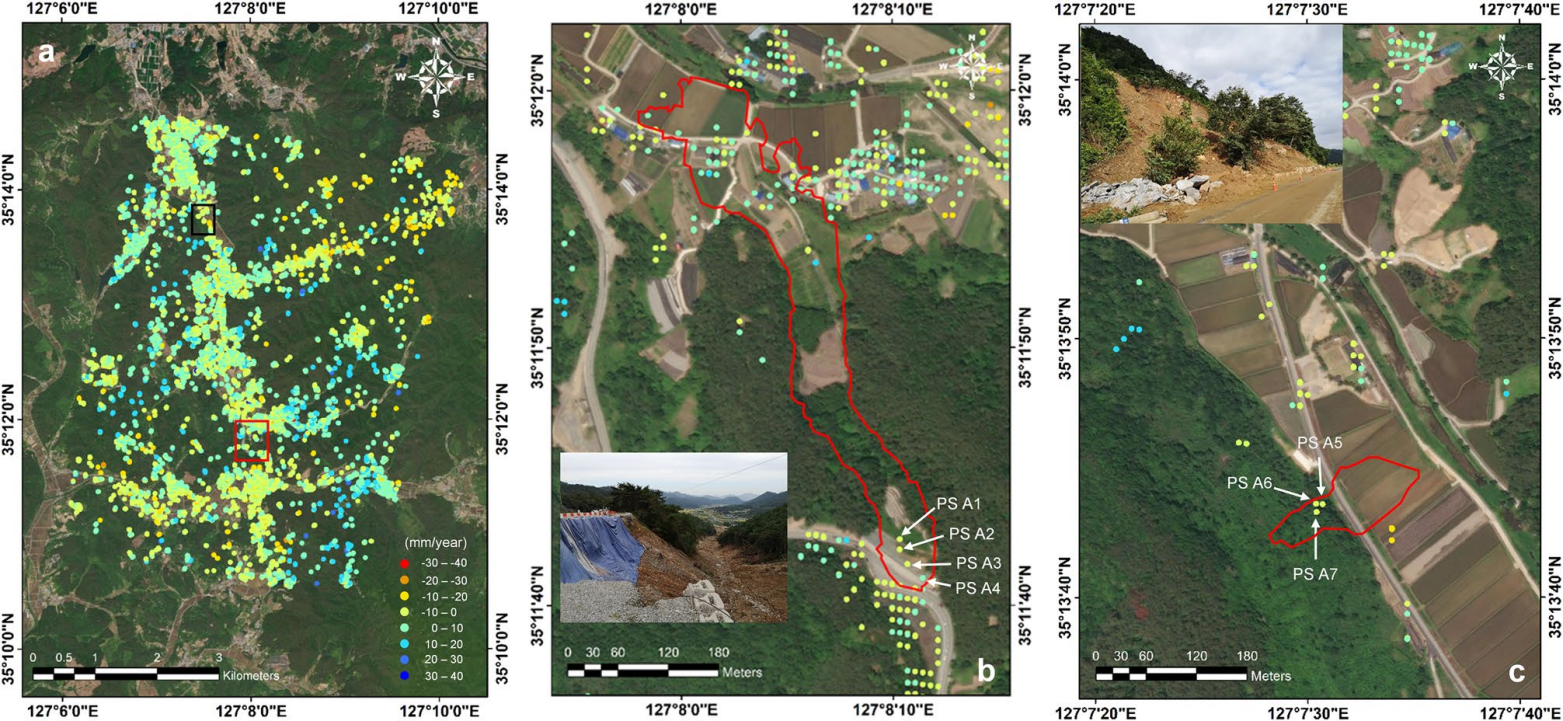
Annual LOS mean velocity map. (**a**) Gokseong landslide site, (**b**) Site 1, and (**c**) Site 2. Note that the red and black rectangles in Fig. 5a indicate the locations of Sites 1 and 2, respectively. Note that the red polygons in Figs. 5b and 5c represent the landslide boundaries. The inset photos show the sites post-landslide.

**Figure 6:**
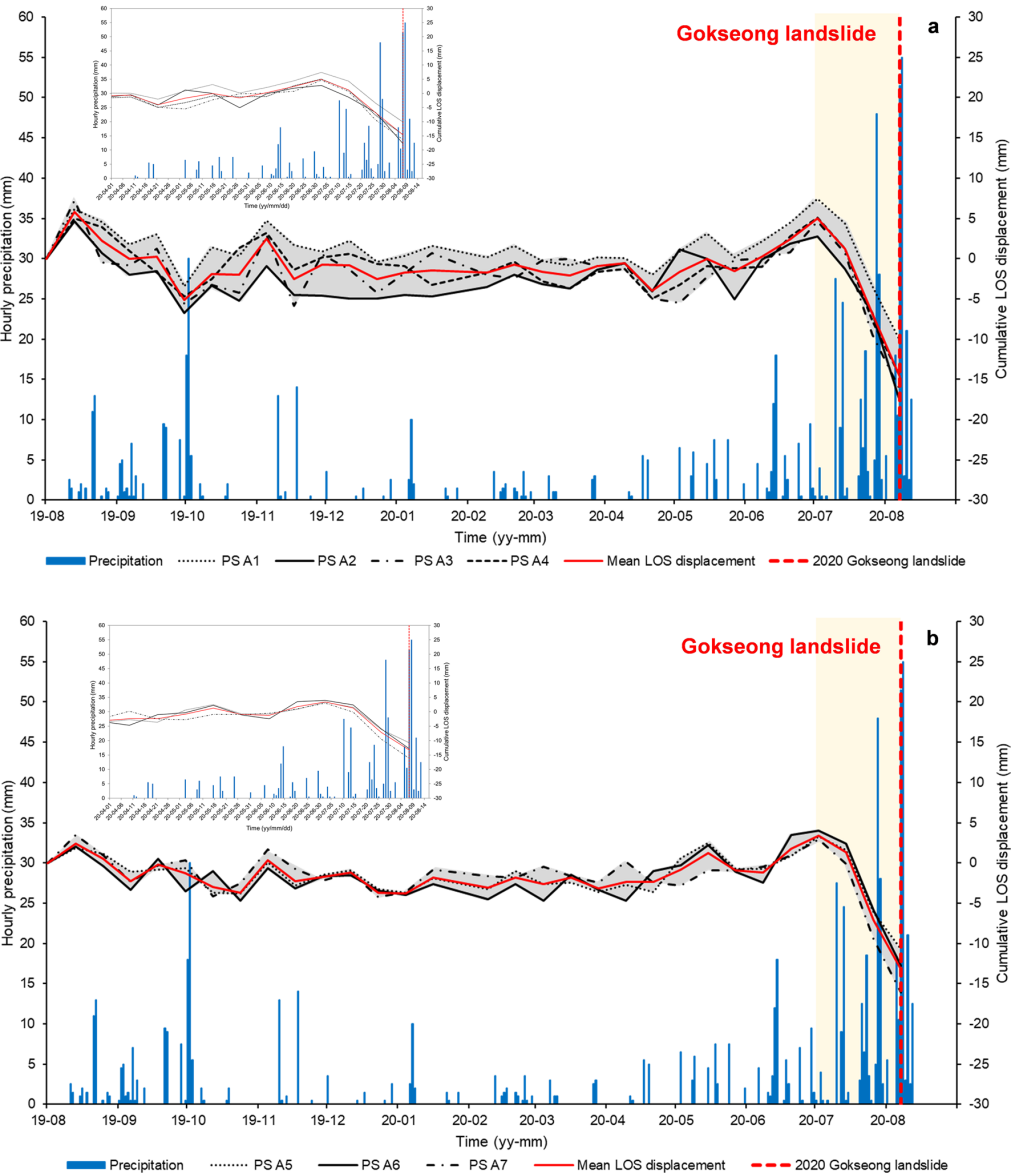
Cumulative LOS displacement in (**a**) Site 1 and (**b**) Site 2. Note that the inset figures represent the results from April to August 2020.

Figure 5 represents the pre-failure annual mean velocity map along the line-of-sight (LOS) direction. Securing observation points in forested areas becomes challenging due to the scattering of radar signals caused by vegetation movement. Fortunately, observation points were obtained on the road near the landslide initiation zone in Site 1 (PS A1-to-A4; Fig. 5b). Figure 6 shows the temporal variations of displacements in the LOS direction, superimposed with hourly precipitation data over time. The LOS displacements were negative, indicating movement away from the satellite along the LOS direction. Prior to the landslide event, the precipitation had continuously influenced the slope movement, specifically during Typhoon Hagupit on August 5, 2020. Similarly, Fig. 5c shows the pre-failure annual mean velocity map and time-series displacement results of a landslide in Site 2, located 4 km away from the Gokseong landslide site. The observed pattern in Site 2 closely resembles that of Site 1 (PS A5-to-A7; Fig. 6b). The displacement was attributed to continuous rainfall that commenced a few days earlier. These findings strongly suggest a significant correlation between landslide occurrences and rainfall patterns. Moreover, the study demonstrates that precise displacement monitoring through satellite InSAR technology can aid in identifying landslide-prone areas and monitoring displacement before major landslides occur.

### Landslide area mapping

The trace of the landslide at Site 1 is illustrated in Fig. 2a. The depth of the eroded channel was approximately 2.5 m. The initiation and deposition zones were located at elevations of 251 m and 160 m above sea level, respectively, with a total landslide runout distance of 678 m. The average slope of the landslide initiation zone was 35°. Additionally, the watershed widths of the initiation and transport zones ranged from approximately 40–60 m, while the maximum width of the deposition fan reached 140 m.

**Figure 7:**
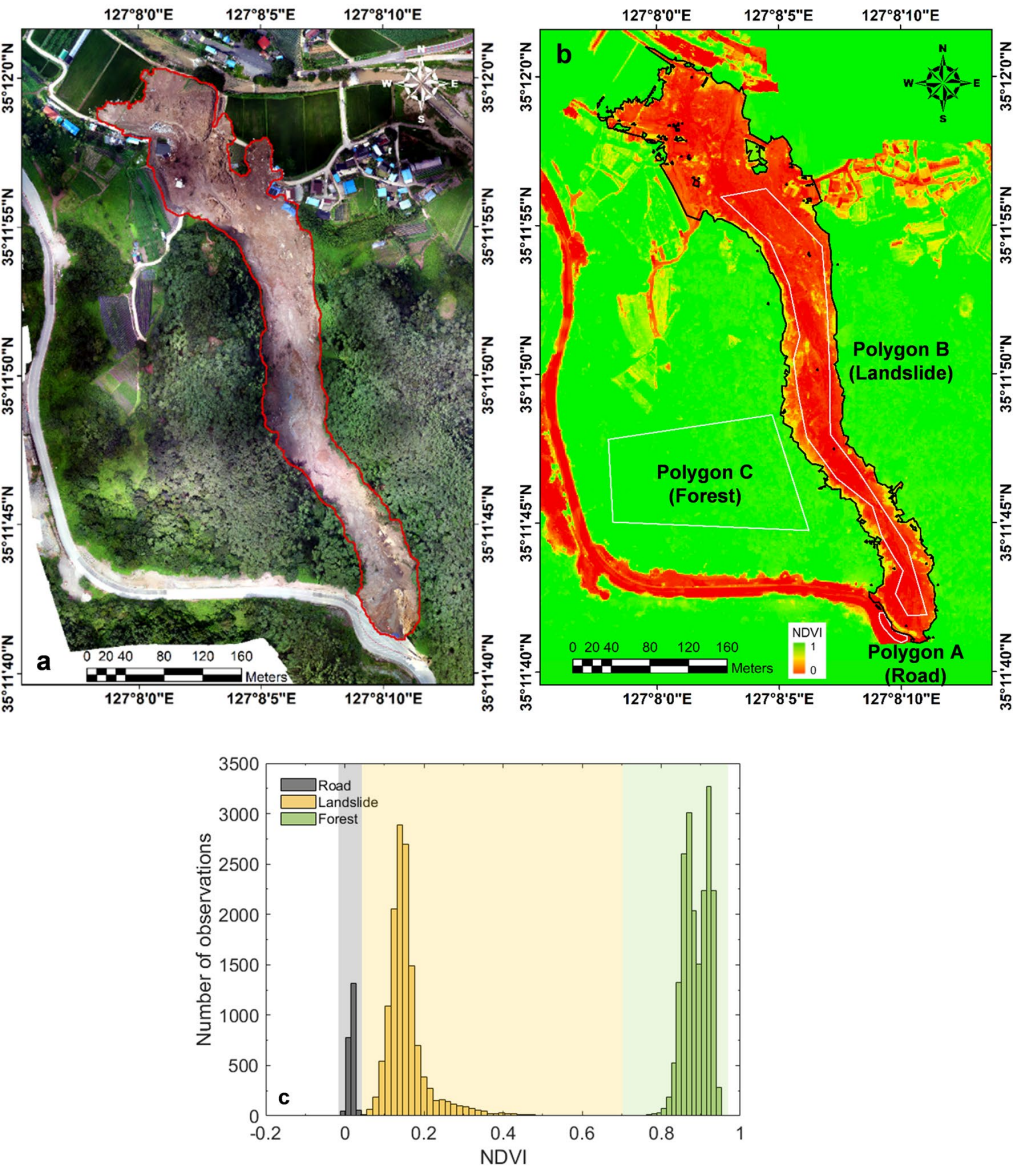
(**a**) RGB composite image (**b**) Spatial distribution of NDVIs obtained from the UAV survey after the Gokseong landslide event and (**c**) NDVI distributions by land cover type. Polygons A, B, and C cover road, landslide, and forest, respectively. Note that the red polygon in Fig. 7a represents the area extracted by manual estimation, the black polygon in Fig. 7b represents the area extracted with an NDVI range 0.04 to 0.70, and the white polygons indicate the sample location to analyze the ranges of the NDVIs.

The NDVI estimated from the multi-spectral data delineated the landslide area (Site 1), as shown in Fig. 7. The range of the NDVI value differed with land-cover types, and Fig. 7c illustrates the NDVI distributions for road, landslide, and forest areas. In this study, the NDVI value of 0.04–0.70 was determined as the landslide area, and as a result, the landslide area was determined to be 4.26 × 10^4^ m^2^. The delineated landslide area well matched with the actual landslide area, highlighting the accuracy of the method employing multi-spectral images, UAV and NDVI.

### Elevation change post-landslide

**Figure 8:**
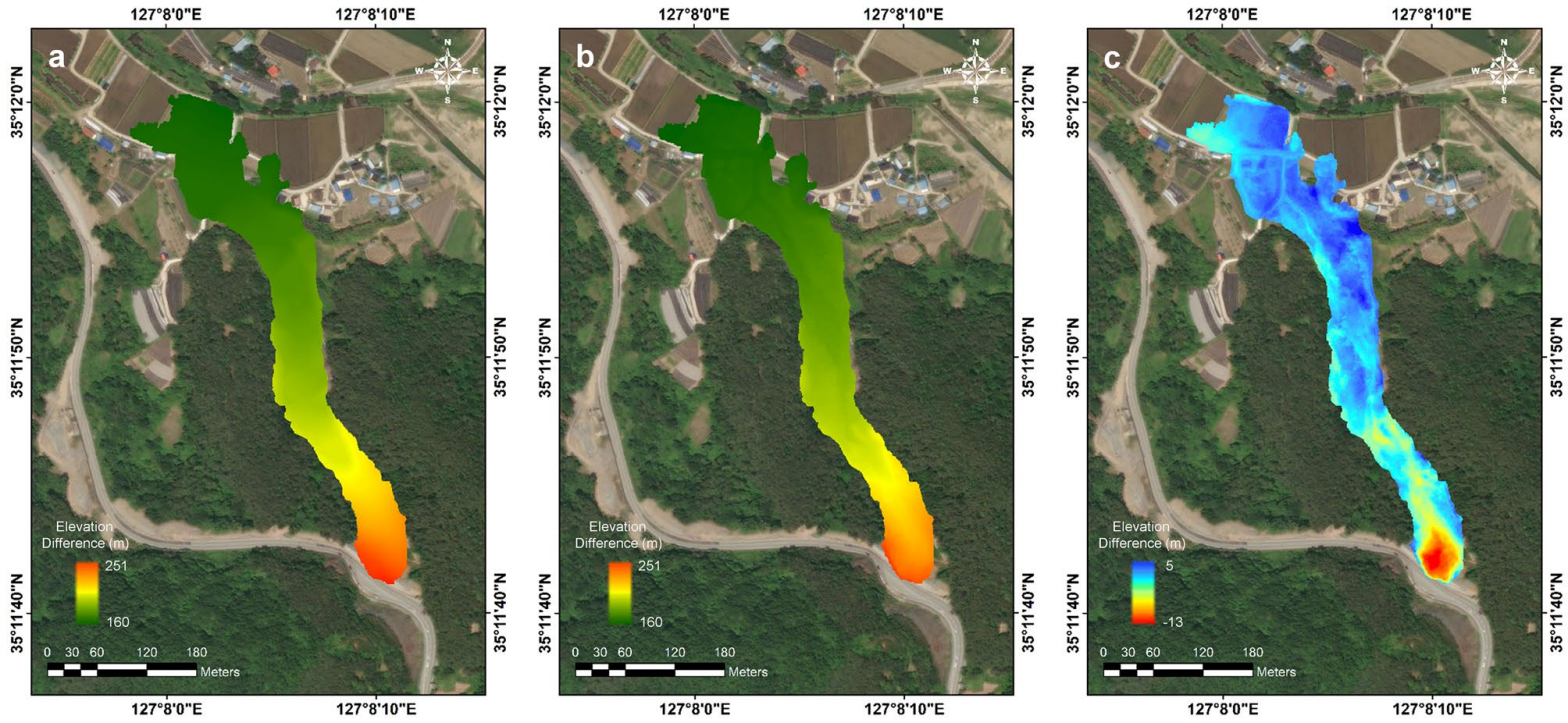
Digital elevation information of the landslide region: (**a**) Before and (**b**) after the event. (**c**) Elevation difference map, which captures the source and deposition areas.

The pre-landslide DEM data was constructed by using the source provided by Korea National Geographic Information Institute (KNGII), as illustrated in Fig. 8a. Following the landslide event, a high-resolution digital elevation model (DEM) of the site was acquired using the UAV-LiDAR system (Fig. 8b). Comparison of these two DEM allowed to identify terrain differences caused by the landslide (Fig. 8c). The negative elevation change indicated the erosion and the positive elevation change means the deposition. The initiation zone of the landslide exhibited a substantial topographic change of more than 13 m. In the downstream area, it was confirmed that a significant amount of debris (5 m in thickness) was deposited as a result of the landslide. For the landslide area derived from the NDVI analysis, the volume of the landslide was calculated based on the changes in the terrain elevation. As a result, the eroded and deposited volumes were estimated to be approximately 5.37 × 10^4^ m^3^ and 1.58 × 10^4^ m^3^, respectively.

## Discussion

### Effect of resolution of the NDVI data on the landslide area and volume

The Normalized Difference Vegetation Index (NDVI) can exhibit variations depending on the timing of data collection. Moreover, NDVI values are subject to change based on the specific characteristics of the area where a landslide has occurred^41–43^. Accurate estimation of the landslide occurrence area requires identifying the appropriate NDVI range. Incorrect selection may result in underestimation or overestimation of the landslide area. Meanwhile, it is worth noting that the resolution of the map heavily affects the determination of NDVI range and landslide areas. Herein, we further compare different data acquisition techniques and examine the effect of image resolution on the results.

**Figure 9:**
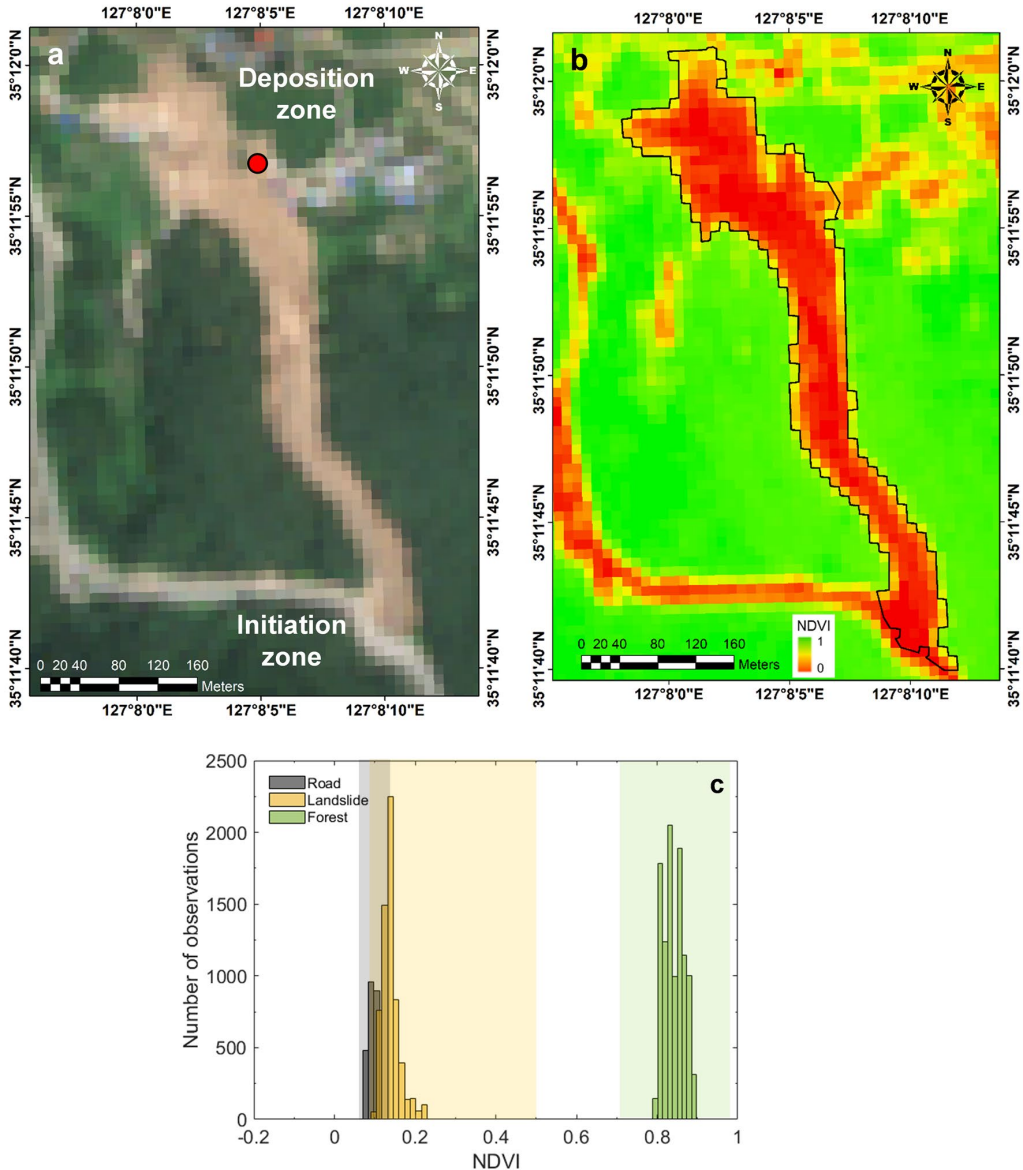
(**a**) Optical image and (**b**) spatial distribution of NDVIs, which were obtained from the Sentinel-2 after the event. **c** Ranges of NDVI by region. Note that the black polygon in Fig. 9b represents the area extracted with an NDVI range 0.08 to 0.53. Note that the red circle indicates the soil sampling point.

**Figure 10:**
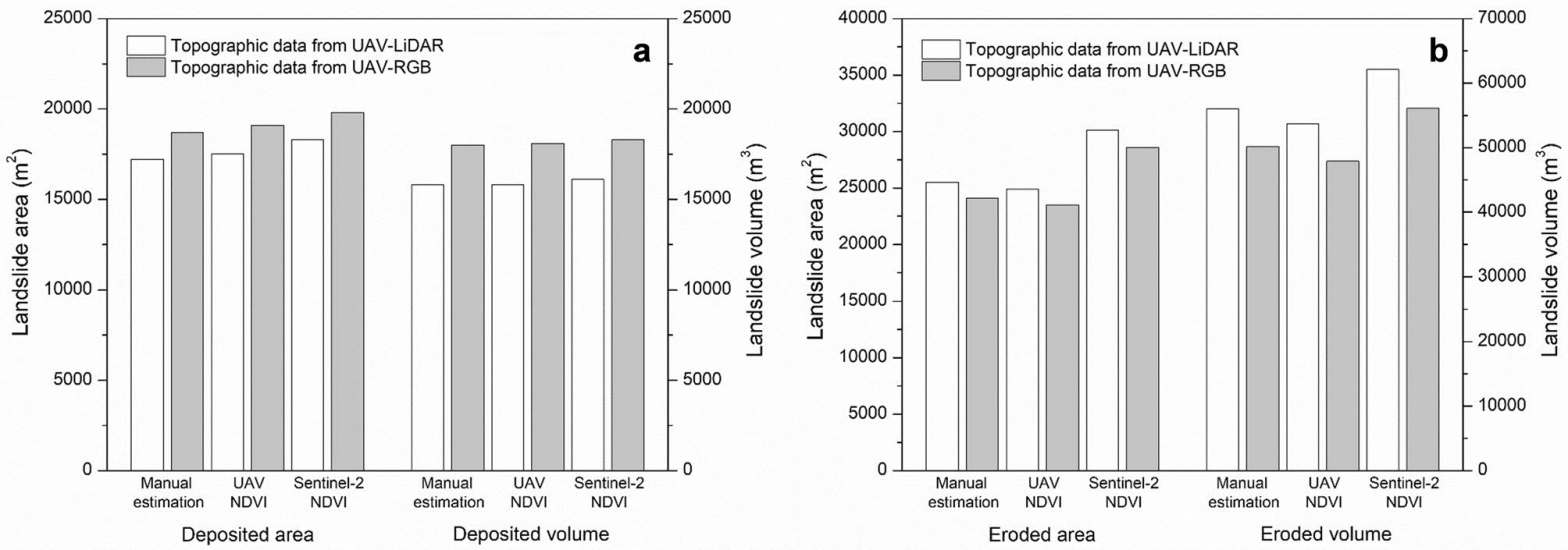
Estimated areas and volumes related to the landslide. (**a**) Results in the deposition zone and (**b**) results in the erosion zone. Note that manual estimation indicates that the landslide area is delineated with visual inspection of the optical image.

This study uses optical and multi-spectral images with 10 m resolution acquired on August 20, 2020 from the Sentinel-2 satellite and obtains an NDVI map (Fig. 9a,b). Herein, the NDVI of 0.08–0.53 is chosen to delineate the landslide area (Fig. 9c). Figures 7b and 9b compare the landslide covers captured from the UAV-driven NDVI map and satellite-driven NDVI map, respectively. The distinction between the road and debris (landslide) boundaries is less clear, especially in the initiation zone, in the satellite-based result compared to the UAV-acquired result. While it is possible to distinguish between the landslide and forest covers, there is an overlapping section between the landslide and the road, as shown in Figure 9c. The image resolution seems to have a minimal impact on the aerial estimates of the depositional area: 1.72 × 10^4^ m^2^ from the UAV-RGB map with the visual inspection method, 1.75 × 10^4^ m^2^ from the UAV-NDVI, and 1.83 × 10^4^ m^2^ from the satellite NDVI, respectively, as illustrated in Fig. 10a. However, it exerts a more significant influence on the erosion area estimation: 2.55 × 10^4^ m^2^ from the UAV-RGB map, 2.49 × 10^4^ m^2^ from the UAV-NDVI map, and 3.01 × 10^4^ m^2^ from the satellite NDVI map (Fig. 10b). These variations can be attributed to the lower resolution of the Sentinel-2 images, resulting in significant overestimation of the erosion area within the landslide region.

Similarly, image resolution has a greater impact on the estimation of erosion volume compared to deposited volume. When the elevation changes acquired from UAV-LiDAR used, the erosion volume is estimated to be 5.60 × 10^4^ m^3^ from the UAV-RGB map, 5.37 × 10^4^ m^3^ from the UAV-NDVI map, and 6.21 × 10^4^ m^3^ from the satellite NDVI map (Fig. 10b). By contrast, the deposited volume appears to be consistent, e.g., approximately 1.58 × 10^4^ m^3^ from the UAV-RGB map, 1.58 × 10^4^ m^3^ from the UAV-NDVI map, and 1.61 × 10^4^ m^3^ from the satellite NDVI map (Fig. 10a).

These results clearly demonstrate that the spatial resolution of NDVI data plays a significant role in determining the area and volume of landslides, particularly in areas with notable topographic changes, i.e., the erosion zone in this study. Therefore, it is crucial to carefully consider and select an appropriate image resolution when conducting landslide investigations to ensure accurate and reliable results.

### Effect of topographic information on the landslide volume

The elevation change can be determined by using two approaches: digital photogrammetry using UAV-RGB images (or UAV-RGB) and 3D LiDAR point cloud (or UAV-LiDAR). In this context, a comparison of these two approaches is conducted, focusing on erosion and deposition volume estimation, as illustrated in Fig. 10. Overall, the UAV-LiDAR method yields a greater erosion volume but a lower deposition volume when compared to the UAV-RGB method. This discrepancy is attributed to the interference of the tree branches in the RGB images. The elevation change near wooded areas is not properly captured in the volume calculation, especially in the narrow upstream area where erosion is prevalent. By contrast, in the downstream area with a wider deposition fan and fewer trees, the difference in deposited volume between the UAV-RGB and UAV-LiDAR methods is relatively minimal (Fig. 10b).

### Distribution of soil water content

The moisture content (or water content) of soil undergoes changes during rainfall infiltration, and hence it is one of the important indicators to rainfall-triggered or rainfall-primed landslides. Specifically, in the event of a landslide and accompanying debris flow, the water contents in the various regions—such as the upslope landslide initiation area, eroded channel bed, and downstream deposition zone—reflects the characteristics of surface soils, including their density and looseness. In this section, the water content of soils at the Gokseong landslide site is estimated using UAV-acquired multi-spectral images. An artificial neural network (ANN) model developed by Lim and his co-workers^44^ is employed for this purpose, which utilizes soil color and NIR reflectance characteristics as input parameters, extracted from the multi-spectral images, to predict the water content of soils.

**Figure 11:**
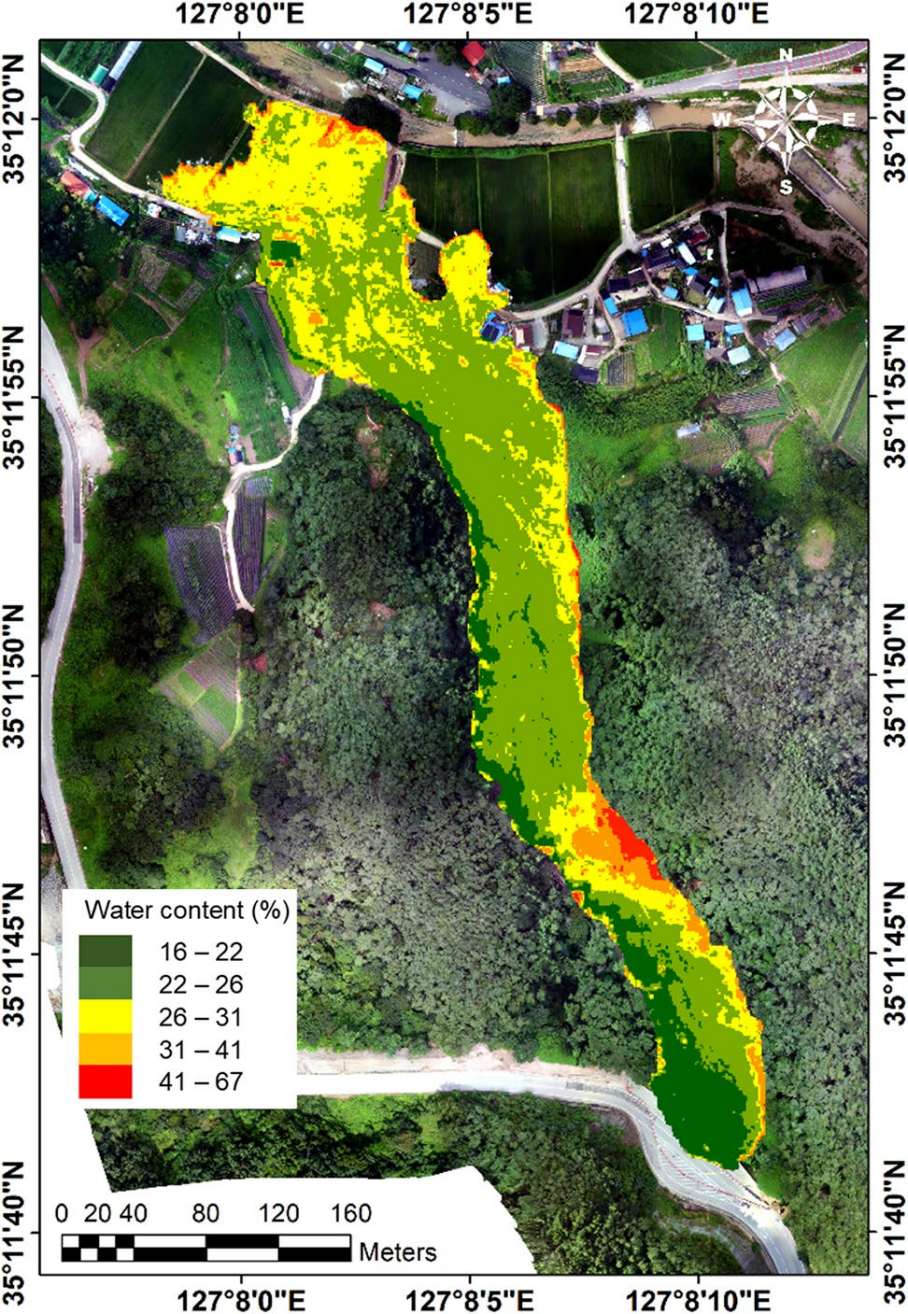
Distribution of soil water content at the landslide site.

Figure 11 illustrates the distribution of soil water content within the soil cover affected by the landslide. The result reveals that the soil in the landslide initiation (source) zone exhibits a low water content, measuring below 22%, while the downstream deposition zone features a higher water content, exceeding 26%. In the initiation zone, the top soil underwent erosion, leaving the exposed soil cover as the original ground. As a result, the soil in this area showed a high compacted density and thus a low water content when fully saturated. Furthermore, the multispectral imaging was carried out a few days after the precipitation ceased, potentially allowing for the drainage of pore water from the steep slope in this region. In contrast, the majority of the soil cover downstream consisted of freshly deposited soil. Consequently, this loosely deposited soil exhibited a higher water content. Along the curved debris flow path, a notable difference in water content is observed between the left and right-side channels due to the prevalence of erosion on one side and the dominance of deposition on the other. Particularly noteworthy is an area in the middle-stream where the estimated soil water content exceeds 41%. This heightened water content is presumed to be primarily a result of substantial soil deposition in this specific corner area. However, it is also worth noting that the shading in this particular region may have influenced the multi-spectral imaging, potentially contributing to this unusually high water content.

To validate the water content estimation based on the ANN model, soil samples were collected from the deposition zone, given restricted access to the landslide site (Fig. 9a). The water content of a sampled soil was measured at 27.8%, while the estimated water content for the corresponding location was 26.5%. Although further validation is required to fully validate the ANN model, the result suggests feasibility of using the multi-spectral images for estimating the water content across large-scale soil covers. The water content data enhances the accuracy of landslide predictions by accounting for the impact of preceding rainfall on landslide occurrence. Furthermore, post-landslide water content data can contribute to improved forecasts of potential collapses.

### Implications of multi-source remote sensing

In this study, we present four remote sensing techniques: satellite-based InSAR, UAV-driven RGB imaging, UAV-driven multi-spectral imaging and UAV-driven LiDAR survey. Before the landslide event, the satellite InSAR technology detects occurrence and continuity of displacement over a wide area. After the landslide event, RGB and multi-spectral image data are used to estimate the extent of the landslide damage area. The eroded and deposited volumes are assessed using topographic data obtained from the UAV-LiDAR system. In addition, the UAV-driven multi-spectral images, in combination with a prediction model, allow estimation of water content of the soil cover. Integration of these valuable data advances our understanding of landslides, and it can facilitate not only prediction of landslide hazard but also planning of effective post-disaster recovery plans.

The satellite InSAR technology plays a crucial role in identifying landslide-prone areas and enables long-term pre-event monitoring, without the need for on-site visits. To ensure high accuracy, it is essential to carefully choose the optimal analysis method among various InSAR methods and related parameters based on the site conditions and type of landslides. In forested regions, the selection of an appropriate radar wavelength for acquiring coherent radar targets becomes especially critical. The radar wavelength directly influences the probability of radar waves being scattered from the crowns or stems of trees. Smaller radar wavelengths tend to increase the likelihood of such scattering occurrences^45–48^.

The UAV-acquiring RGB imaging offers numerous advantages in various applications. One significant benefit is the capability to acquire a digital surface model (DSM). Moreover, it facilitates visible inspections for landslide triggers without the need for on-site access. Additionally, the UAV-acquiring RGB imaging proves valuable in manually estimating the extent of landslides, providing a means to cross-verify results obtained from the NDVI method. Furthermore, this UAV-acquiring RGB imaging technology demonstrates remarkable efficiency and rapidity in monitoring areas with minimal vegetation or exposed terrain, such as rocky mountain, post-landslide sites, and bare soil. The simplicity of operating UAVs and processing data makes it an optimal choice for such monitoring tasks. However, it is important to note that in regions with dense vegetation, the UAV-LiDAR system becomes indispensable for acquiring accurate topographic information. The UAV-LiDAR technology offers a significant advantage by providing topographic information even in densely vegetated areas. However, LiDAR sensors using specific wavelengths may encounter limitations in data collection when the ground is saturated. In this study, the LiDAR points were not acquired for five days after the Gokseong landslide event, as the soil remained saturated after the event (the LiDAR sensor operated at a wavelength of 905 nm in this study). Fifteen days after the landslide event, the soil had dried sufficiently to obtain LiDAR points. The selection of appropriate LiDAR sensors is crucial, especially when dealing with monitoring tasks in areas with saturated ground shortly after a landslide event.

## Conclusions

This study presents a comprehensive demonstration of the multi-source remote sensing technology employed to analyze the Gokseong landslide in South Korea. The novel approach involved utilizing UAV-mounted RGB, multi-spectral, and LiDAR sensors, and satellite SAR sensor. The key findings derived from this study are as follows:

- The research employed satellite InSAR technology to monitor ground displacement before the occurrence of the landslide. The satellite InSAR technology can provide time-series displacement of the study area, which is critical in understanding the pre-landslide displacement patterns. The displacement persisted prior to the landslide, and its pattern exhibited a significant correlation with rainfall in the region. The selection of radar wavelength and InSAR analysis methods should be considered concerning the type of landslides and field characteristics.
- The UAV equipped with RGB and multi-spectral sensors offer a valuable means of acquiring precise information regarding the topography and land-cover of the study area. The UAV-mounted RGB, multi-spectral sensors can help identify traces and erosion patterns of the landslide. The landslide area analyzed using the NDVI was consistent with the results obtained from the manual estimation.
- The landslide volume was analyzed by acquiring topographic information through the UAV-LiDAR technology. Although the flight and processing procedures are relatively complex compared to the UAV-RGB technology, this method has the distinct advantage of collecting topographic information in forested areas. LiDAR data allows for precise capturing of the topography and provides high-resolution elevation information.
- The multi-source remote sensing technology can provide a comprehensive understanding of landslide characteristics, significantly enhancing disaster risk assessment and aiding in the formulation of effective recovery plans.

## Data Availability

The data and materials used in this article are available upon request by the correspondence author.

## Abbreviations

LiDAR: Light detection and ranging
UAV: Unmanned aerial vehicle
InSAR: Interferometric synthetic aperture radar
RGB: Red, green, blue
Radar: Radio detection and ranging
IR: Infrared radiation
KMA: Korea Meteorological Administration
SNAP: Sentinel application platform
PS-InSAR: Permanent scatterer InSAR
NIR: Near-infrared
SfM: Structure from motion
GCP: Ground control point
GNSS: Global navigation satellite system
NDVI: Normalized difference vegetation index
IMU: Inertial measurement unit
M3C2: Multiscale model-to-model cloud comparison
LOS: Line of sight
KNGII: Korea National Geographic Information Institute
DEM: Digital elevation model
